# An Interesting Association of Cystic Hygroma of the Neck and Lymphangioma Causing a Paediatric Swollen Tongue

**DOI:** 10.1155/2016/7930945

**Published:** 2016-03-16

**Authors:** A. N. Beech, J. N. Farrier

**Affiliations:** Department of Oral and Maxillofacial Surgery, Gloucestershire Royal Hospital, Gloucestershire Hospitals NHS Foundation Trust, Great Western Road, Gloucester GL50 3DU, UK

## Abstract

Up to 75% of lymphatic malformations occur in the head and neck region. Of these, cystic hygromas and lymphangiomas have been widely reported; however they rarely occur in the same patient. We report the case of a 5-year-old girl who presented to the Department of Paediatrics of a district general hospital with a short history of recurrent, painful swelling of the anterior one-third of her tongue. She was reviewed under the joint care of the Oral and Maxillofacial Surgery and Otolaryngology Teams. Relevant past medical history included a previously excised cystic hygroma from her right neck when she was aged 2 years. Diagnosis of lymphangioma was made and of the potential management options available active monitoring was favoured due to the patient's age. To our knowledge the occurrence of both tongue lymphangioma and cystic hygroma has not been previously reported in a paediatric patient. This case report therefore shows a rare association between a cystic hygroma of the neck and lymphangioma of the tongue.

## 1. Introduction

Lymphangiomas and cystic hygromas are types of lymphatic malformations. These conditions have a higher propensity for presentation in the head and neck as opposed to other sites, and it is thought that up to 75% of these are found in the oral cavity [1]. The majority of these lymphatic malformations present either at birth or before 2 years of age with between 40 and 50% involving the dorsum of the tongue [2, 3]. The likelihood that two lymphatic malformations could exist in the same patient is possible and has been reported previously [4]. The incidence of this association however is unclear. Because of the rarity of both of these conditions separately, this case report describes the association of tongue lymphangioma with a previously excised cystic hygroma in the same patient.

## 2. Case Report

**Figure 1:**
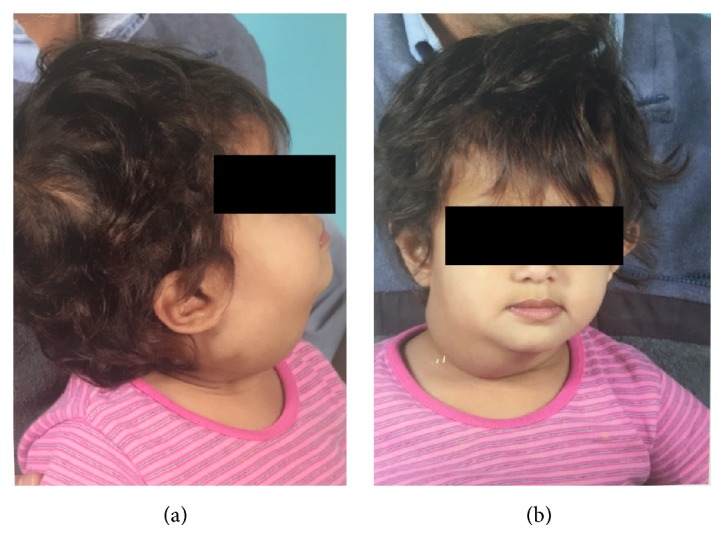
Preoperative clinical photographs of previously excised cystic hygroma in the right neck.

A 5-year-old girl presented to the Department of Paediatrics following referral from her general medical practitioner. She was then referred to the joint care of the Oral and Maxillofacial Surgery and Otolaryngology teams. The patient's mother gave a short history of recurrent and painful swelling of the tongue which was managed by simple analgesia. Sensation and taste were normal. There was no weight loss and no airway obstruction. The patient was medically fit and well with the only medical history of note being the excision of a cystic hygroma from the right neck under general anaesthetic (Figures 1(a) and 1(b)) when the patient was 2 years old. There was no family history of a similar diagnosis or presentation.

**Figure 2:**
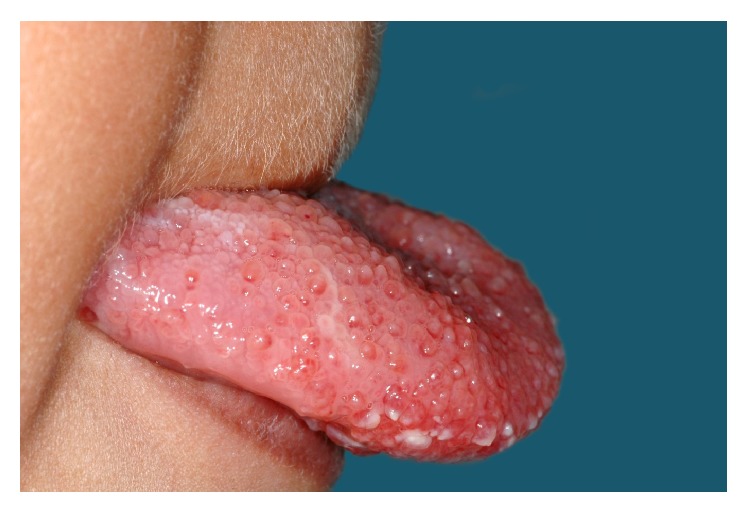
Clinical photograph of grossly enlarged papillae on the anterior one-third of the tongue.

**Figure 3:**
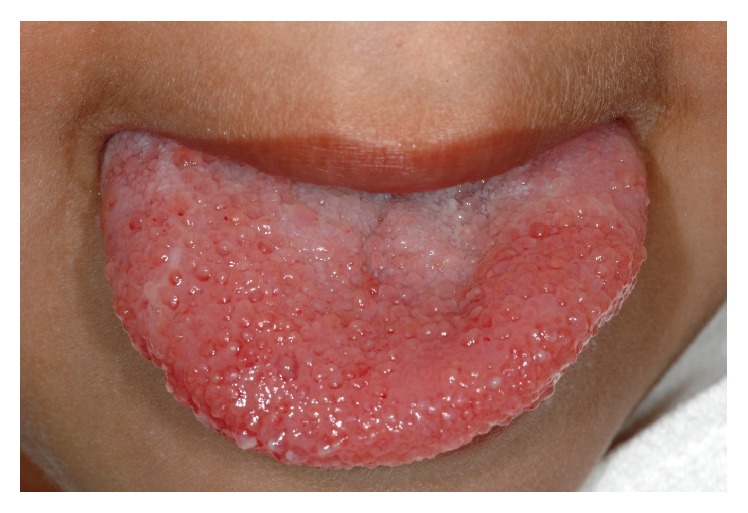
Clinical photograph of grossly enlarged papillae on the anterior one-third of the tongue.

On clinical examination there was a bilateral erythematous swelling of the anterior one-third of the dorsum of the tongue which was soft, but tender on palpation. The surface papillae were grossly enlarged (Figures 2 and 3). The floor of the mouth was unaffected. There were no palpable lymph nodes in the neck and no other swellings evident elsewhere. The surgical site in the right neck was well healed.

Provisional diagnosis was thought to be either a nutritional deficiency, for example, iron deficiency, an infective cause, for example, herpes virus, a bacterial cause, for example, streptococcal infection, a fungal cause, for example, candida or other general conditions, for example, hypothyroidism, diabetes, or pemphigus. Lymphangioma was also in the list of provisional diagnoses. A consideration in an older patient could include prescription drugs, for example, adverse reaction to ACE inhibitors.

The patient had a full set of haematinic tests taken including a full blood count, C-reactive protein, and an autoantibody screen, all of which returned within normal ranges. She also had an ultrasound scan of her tongue from a submental approach which showed no obvious abnormality of the tongue. Because of this no further imaging was deemed necessary on a patient of this age. A biopsy was performed under a general anaesthetic and features were reported as entirely consistent with a clinical diagnosis of a simple/capillary lymphangioma of the tongue.

Over a 3-year period the patient presented episodically with increased tongue swelling to the Paediatric Inpatient Ward. After initial presentation she presented again at 9 months, 21 months, and 35 months. During these inpatient stays the required management included intravenous steroids and analgesia. On each occasion the patient was discharged after resolution of her symptoms 2–4 days later.

Due to the patient's age and the diffuse nature of the lymphangioma conservative management was deemed appropriate in the form of active monitoring. Clinically there had been no other obvious lymphatic malformations or any indication of multifocal disease during the patient's 3 years of care to indicate that full-body scanning would be required. The patient was ultimately discharged from the care of the Oral and Maxillofacial Surgery Department after satisfactory review 1 year following her final inpatient admission.

## 3. Discussion

Lymphangiomas are a group of benign lymphatic malformations with a common predilection to the head and neck region [1] in 50–70% of cases [5]. However other sites such as the mediastinum, axilla, retroperitoneal area, and pelvis have been described in the available literature [6, 7]. The aetiology of these cystic lesions remains unclear, but common suggestions are arrest of lymphatic growth, failure of the lymphatic system to reach the venous drainage, or malplacement of lymphatic channels during embryogenesis [8].

The incidence of lymphangiomas is reported to be 1.2–1.8 per 1000 of new births [9] or 1 in 2000–4000 live births [10] in different studies. They occur in 90% of reported cases between birth and two years of age [11]. However around 100 cases have been reported in adults with the development of the lymphangiomas being secondary to local trauma [12, 13], inflammation, or infection [13].

Cystic hygromas form the majority of head and neck lymphatic malformations and 75% are located in the head and neck region [14]. In one study a link was found between cystic hygromas and chromosomal abnormalities. Of the 58 cases studied 21% of patients had Turner syndrome whilst 25% had Down's syndrome [15]. There was no known chromosomal abnormality in the case we report.

Cystic hygroma presents most commonly as a mass or swelling which is often painless and ill defined, is not attached to overlying skin or mucosa, and can be transilluminated. Obstructive symptoms such as respiratory distress and dysphagia can occur with large masses especially those in the suprahyoid region. Both anterior and posterior triangles of the neck can be involved; however anterior triangle masses are more predominant [16].

As in this case, most head and neck lymphangiomas present at a later stage to cystic hygromas. Patients may present with macroglossia, dysphagia, and an element of airway obstruction. Other common presenting complaints include failure to thrive and sleep apnoea which are more noticeable in newborns. Clinical examination of tongue lymphangioma, because of the extreme swelling often present, usually reveals that tongue papillae have a clear, irregular, and translucent vesicular appearance. This is well represented by this case's clinical photographs.

For large suspected lymphatic malformation a mode of radiological imaging is important in diagnosis and treatment planning [17]. An ultrasound is often sufficient and certainly the most appropriate modality in young patients in terms of compliance; it also avoids further anaesthetics to perform any further scanning, that is, MRI or CT. An MRI scan is indicated, and preferable to CT scanning, if sufficient information is not gained using ultrasound.

The patient had 2 separate lymphatic malformations and was therefore at risk of having additional multifocal disease. This can be confirmed by performing a full-body MRI scan. At each presentation during 3 years the patient showed no obvious signs of any additional lesions and further scanning was deemed unnecessary.

Management of hygromas is usually surgical but can be dependent on size and location and the impact that such surgery could have on local vital structures and on quality of life. Suprahyoid lesions are associated with a higher recurrence rate, morbidity, and surgical complications than infrahyoid lesions [18]. Recurrence is also associated with multiple lesions, those in the midline and incomplete surgical excision. At the age of seven, at final review of her tongue lymphangioma, this young girl showed no signs of recurrence of the previously excised hygroma of her right neck.

Lymphangiomas can be more difficult to manage than hygromas. Whilst surgical excision is an option in larger cases of lymphangioma [19, 20], it can be difficult due to their diffuse nature. Surgical excision is therefore often incomplete. There have been documented cases that have shown varying success with sclerotherapy using intralesional sirolimus [21], bleomycin [22], OK-432—a strain of Group A* Streptococcus* [23], and doxycycline, ethanol, and hypertonic glucose [24] in adults.

Studies specifically in children with lymphatic malformations have shown good success rates with image guided sclerotherapy using doxycycline [25], doxycycline combined with sodium tetradecyl sulfate [26], and 98% ethanol insertion [27]. These studies demonstrated good radiographic resolution rates on the nonsurgical treatment of macrocystic lymphatic malformations, for example, cystic hygromas ranging between 87.5% and 95.2%. Despite containing relatively low patient numbers, there were very few complications observed. All studies, however, reported little success on microcystic/simple malformations, for example, lymphangioma. There was also no testing on intraoral malformations.

Some cases of lymphangioma can resolve spontaneously, as in the case of this young girl. Consideration was given to the available topical or systemic therapies in this case, but, with their varied success, the age of the patient and gradual reduction in frequency of events active monitoring were favoured. This proved to be an acceptable form of management for this patient; however had episodes become more frequent or prolonged then further consideration would have been given to these additional management options.

## 4. Conclusion

Full investigation into abnormalities and swellings of the tongue should be undertaken before diagnosis, as well as subsequent management, of lymphangioma. In this case the history of another lymphatic malformation, a previously excised cystic hygroma, increases the likelihood of lymphangioma, but this is still rare. Whilst surgical excision of a cystic hygroma is most commonly excision the management of a lymphangioma is more challenging. Lymphangiomas are diffuse lesions making surgical excision difficult and potentially disfiguring. The antisclerosing agents can lead to significant morbidity and have had shown mixed success; therefore a “watch and wait” policy is often the best option in the young, as demonstrated in this case report.
